# Left Superior Homonymous Quadrantanopia due to Giant Virchow-Robin Space

**DOI:** 10.1159/000525899

**Published:** 2023-07-03

**Authors:** Michelle Lim, Jonathan A. Micieli

**Affiliations:** a Faculty of Medicine, University of Toronto, Toronto, ON, Canada; b Department of Ophthalmology and Vision Sciences, University of Toronto, Toronto, ON, Canada; c Kensington Vision and Research Centre, Toronto, ON, Canada

**Keywords:** Hemianopia, Virchow-Robin space, Vision loss

## Abstract

A 51-year-old man was referred by his optometrist for an incidental finding of a visual field defect. Humphrey 24-2 SITA-Fast visual field testing revealed a left superior homonymous quadrantanopia, and magnetic resonance imaging of the brain showed a 2.0 × 0.5-cm oblong-shaped cerebrospinal fluid space posterior to the right basal ganglia. This space coursed close to the lateral geniculate body and was thought to represent a giant perivascular (Virchow-Robin) space. This case demonstrates that patients with a visual field defect without other neurological symptoms could be a result of an enlarged Virchow-Robin space along the visual pathway.

## Introduction

Virchow-Robin spaces are perivascular compartments that surround arteries perforating the subarachnoid space through the brain [1]. While small Virchow-Robin spaces are present in all age groups, they can enlarge with advancing age [2]. On MRI sequences, these enlarged spaces are visualized as hyperintensities on T2-weighted sequence [3]. Enlarged Virchow-Robin spaces are usually benign and do not cause severe developmental symptoms [4]. In some cases, they can result in hypertension, positional vertigo, headache, or hemisensory disturbances [4, 5]. Few studies have reported patients presenting with visual field disturbances [6–8]. Here, we describe a case of left superior homonymous quadrantanopia where the patient was found to have a giant Virchow-Robin space in the right basal ganglia.

## Case Report

**Fig. 1.**
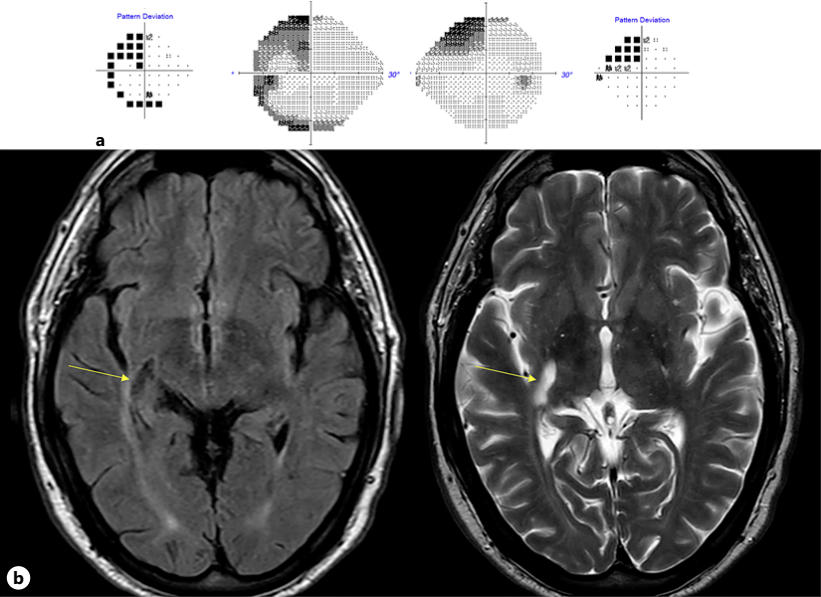
**a** Humphrey 24-2 SITA-Fast visual field testing showing a left superior homonymous quadrantanopia. **b** MRI FLAIR (left) and T2 demonstrating a 2.0 × 0.5-cm oblong-shaped cerebrospinal fluid-filled space posterior to the right basal ganglia, just above the temporal horn of the lateral ventricle, which courses close to the expected location of the lateral genicular body. A thin rim of FLAIR hyperintensity is noted at the margin of the lesion. This was thought to represent a giant Virchow-Robin space.

A 51-year-old man was referred from his optometrist for a visual field defect. He had a past medical history of type 2 diabetes, hypertension, and gastroesophageal reflux disease. His medications included metformin, rosuvastatin, telmisartan, and pantoprazole. He was asymptomatic and went to see his optometrist for a routine exam. He was found to have a left homonymous visual field defect and was referred to neuro-ophthalmology. Neuro-ophthalmic examination revealed a visual acuity of 20/20 OD and 20/20 OS, color vision was normal, and there was no relative afferent pupillary defect. Dilated fundus examination was normal. Humphrey 24-2 SITA-Fast visual field testing revealed a left superior homonymous quadrantanopia (shown in Fig. 1a). Magnetic resonance imaging of the brain was performed and showed a 2.0 × 0.5-cm oblong-shaped cerebrospinal fluid space posterior to the right basal ganglia, which coursed close to the expected location of the lateral geniculate body (shown in Fig. 1b). This was thought to represent a giant perivascular (Virchow-Robin) space. Given that the patient was asymptomatic, no intervention or neurosurgery consultant was recommended. His visual field was stable at the 1-year follow-up.

## Conclusion

Our report describes a rare case of a patient presenting with left superior homonymous quadrantanopia and was found to have a giant Virchow-Robin space posterior to the right basal ganglia. To the best of our knowledge, only three cases of visual field defects associated with giant Virchow-Robin spaces have been reported. Buerge et al. [7] reported a 77-year-old woman presenting with progressive cognitive decline, left hemianopsia, and left pyramidal signs. MRI revealed enlarged Virchow-Robin spaces in the right centrum semiovale and right temporo-occipital white matter. Zafar et al. [8] described a 55-year-old woman presenting with left intermittent hemianopsia, memory disturbances, and vomiting. MRI showed a large Virchow-Robin space near the right optic radiation. Lastly, Rivet et al. [6] described a 53-year-old woman with a left inferior homonymous quadrantanopia and mild left arm weakness. MRI disclosed a giant Virchow-Robin space compressing the right optic tract. While patients in previous cases experienced neurological signs and symptoms, visual field defects were the only presenting symptom in our patient and he had a normal neurological examination. This case demonstrates that patients with a visual field defect without other neurological symptoms could be a result of an enlarged Virchow-Robin space along the visual pathway.

## Statement of Ethics

This patient has given his written informed consent to publish the case (including publication of images). Written informed consent was obtained from the patient for publication of the details of their medical case and any accompanying images. Research ethics board approval was not required from the University of Toronto Research Ethics Board. Ethical approval is not required for this study in accordance with local or national guidelines.

## Conflict of Interest Statement

The authors have no conflicts of interest to declare.

## Funding Sources

No funding was received for this study.

## Author Contributions

Conception and design, and final approval (Michelle Lim and Jonathan A. Micieli); data collection and preparation of manuscript (Michelle Lim); and critical revisions (Jonathan A. Micieli).

## Data Availability

All data generated or analyzed during this study are included in this article. Further inquiries can be directed to the corresponding author.
